# Physical exercise and cognitive engagement outcomes for mild neurocognitive disorder: a group-randomized pilot trial

**DOI:** 10.1186/s13063-018-2865-3

**Published:** 2018-10-19

**Authors:** Liselotte De Wit, Deirdre O’Shea, Melanie Chandler, Tripti Bhaskar, Jared Tanner, Prashanthi Vemuri, Julia Crook, Miranda Morris, Glenn Smith

**Affiliations:** 1 Department of Clinical and Health Psychology, University of Florida, 1225 Center Drive, Gainesville, FL 32606 USA; 2 Institutional address: Department of Psychiatry and Psychology, Mayo Clinic, 4500 San Pablo Road, Jacksonville, FL 32224 USA; 3 Tallahassee Memorial HealthCare, 1401 Centerville Road, Tallahassee, FL 32308 USA; 4 Department of Radiology, Mayo Clinic, 200 1st street SW, Rochester, MN 55905 USA

**Keywords:** Mild cognitive impairment, Neurocognitive disorder, Behavioral interventions, Cognitive training, Yoga

## Abstract

**Background:**

Amnestic mild cognitive impairment (aMCI) is considered a risk state for the development of dementia due to Alzheimer’s disease. It is also a period in which interventions may be most effective in slowing progression to dementia. Computerized cognitive training and increased physical activity have shown to be among the most promising interventions. However, current evidence from randomized controlled trials comparing cognitive training, physical activity, and an active control is inconsistent. Furthermore, the neural mechanisms underlying these interventions are currently unclear.

**Methods:**

The objective of the current pilot study is to explore the feasibility of a trial investigating the impact of computerized cognitive training, yoga, and an active control intervention (wellness education) in individuals with aMCI by conducting a group-randomized, multisite, parallel, three-arm pilot study. We will establish preliminary effect sizes regarding the association of each intervention with neuroimaging and cognitive and participant-reported measures. We also aim to estimate the strength of association between the various outcomes. The current trial aims to recruit 75 people with aMCI and their 75 cognitively healthy care partners through clinics and senior care facilities. The initial intervention will last 10 days and will consist of 1 h daily of the assigned intervention i.e., (yoga, computerized cognitive training, or wellness education) combined with 1 h of memory compensation training and 1 h of support groups. Twenty-five participants will be group-randomized to each arm using a random number generator. Study staff and participants will be kept blind until recruitment is complete for each group. After the initial two-week intervention, participants will continue the assigned intervention for 24 weeks. Outcome measures are: functional connectivity and cerebral perfusion as assessed by magnetic resonance imaging; cognition; daily functioning; mood; anxiety; self-efficacy; caregiver burden; quality of life; and study feasibility including recruitment and retention rates.

**Discussion:**

This pilot trial aims to investigate the feasibility of a trial studying the impact of computerized cognitive training, yoga, and an active control intervention in persons with aMCI on MRI-based functional connectivity and cerebral perfusion as well as cognition, daily functioning, mood, anxiety, and quality of life and feasibility?

**Trial registrations:**

ClinicalTrials.gov, NCT03095170. Registered on 23 March 2017.

**Electronic supplementary material:**

The online version of this article (10.1186/s13063-018-2865-3) contains supplementary material, which is available to authorized users.

## Background

Mild cognitive impairment (MCI) is often considered a risk state, or even a prodromal stage, for the development of dementia. MCI is characterized by the following criteria: (1) a cognitive concern; (2) cognitive impairment as measured by psychometric tests; (3) overall intact activities of daily living (ADLs); and (4) not meeting criteria for dementia [1]. In the Diagnostic and Statistical Manual of Mental Disorders, 5th Edition, the concept of MCI is named “Minor Neurocognitive Disorder” [2]. Delaying the progression to dementia in patients with MCI would drastically impact the economic burden of dementia [3] as well as the quality of life of patients with MCI and their families. For these reasons, there has been increasing interest in interventions and rehabilitation programs that target MCI.

The National Academy of Medicine brought together a committee of experts on Preventing Dementia and Cognitive Impairment to objectively document an evidence-based consensus on dementia and cognitive impairment research. Their findings, reported in *Preventing Cognitive Decline and Dementia: A Way Forward* [4], indicated that cognitive training and increased physical activity are promising interventions to prevent, delay, or slow MCI or clinical Alzheimer’s-type dementia. However, the current evidence from randomized controlled trials is insufficient. To improve future research and obtain more evidence on these interventions, they suggested methodological improvements such as using biomarkers and having an appropriate control group. They also indicated that most studies, thus far, have focused on individual interventions while a multi-modal model may be more beneficial.

The current trial, called Physical Exercise And Cognitive Engagement Outcomes for Mild Neurocognitive Disorder (PEACEOFMND), aims to incorporate these methodological suggestions. The PEACEOFMND trial will investigate the impact of computerized cognitive training versus yoga versus an active control group (wellness education) on functional connectivity and cerebral perfusion in the brain as well as on cognitive functions (working memory, attention, and global cognitive functioning), daily functioning, mood, anxiety, self-efficacy, caregiver burden, and quality of life. Associated with these behavioral interventions will also be assessed. To create a multi-component program, each experimental arm is combined with support groups and memory compensation training (MCT). Individuals with MCI and caregivers that participate in group therapy show greater acceptance of the MCI diagnoses than wait-list controls [5]. Memory compensation training was developed for individuals with *amnestic* MCI (aMCI) and involves training the use of a portable calendar and note-taking [6]. Individuals with MCI that are compliant in MCT are found to have a higher sense of memory-related self-efficacy and functional ability [7]. Other multicomponent intervention trials have shown to improve or maintain cognitive functioning [8]. Still, the neuroimaging correlates associated with these program elements have not been investigated thus far.

With regards to computerized cognitive training, the primary neuroplasticity changes have been observed by the use of functional MRI (fMRI) [9, 10]. Previous research has found that computerized cognitive training improvements in problem solving and reasoning were related to changes in occipitotemporal white matter integrity and functional connectivity between the superior parietal cortex and the inferior temporal lobe [9, 10]. It is believed that computerized cognitive training may help stabilize, recover, or compensate for loss of functional and structural brain connectivity due to the ongoing pathological processes in aMCI.

Physical exercise has also been suggested as a promising intervention to reduce cognitive decline in older adults at risk for dementia [11]. Physical exercise as a part of a multi-component intervention has shown to slow cognitive decline [8] and is associated with higher gray matter volume in MCI [12]. There is currently insufficient evidence to determine the most beneficial type of exercise for decelerating the rate of cognitive decline and dementia [4]. Yoga incorporates aerobic effects, strength, flexibility, and balance. Aerobic physical exercise is thought to improve cerebral perfusion mechanisms [13–15]. Aerobic exercise [16, 17] and flexibility, toning, and balance training [17] have also shown to impact functional connectivity in older adults and MCI.

The design of the current trial is a group-randomized, multisite, parallel, three-arm trial that seeks to compare the impact of the following interventions: (1) computerized cognitive training; (2) yoga; and (3) an active control (wellness education).

### Trial objectives and hypotheses

The primary aims of this pilot are:

#### Aim 1

To conduct a preliminary examination of the impact of computerized cognitive training, yoga, and an active control intervention (wellness education) on changes in cerebral perfusion and functional connectivity, as measured by magnetic resonance imaging (MRI) in people with aMCI. Specifically, we aim to:

- **1A:** Estimate effect sizes for changes in cerebral perfusion and functional connectivity in the different treatment groups. We hypothesize that there will be preliminary evidence for improved functional connectivity in the computerized cognitive training group compared to the other two groups and that there will be changes in the cerebral perfusion as a result of yoga.
- **1B:** Estimate the relationships between changes in cerebral perfusion and functional connectivity, cognitive function, daily function, mood, anxiety, and quality of life within the two experimental groups compared to the control group. We hypothesize that improvement in cerebral perfusion and functional connectivity will be associated with improvement in cognitive functions, daily function, mood, anxiety, and quality of life in the individuals with MCI. Furthermore, we hypothesize that these changes are associated with an improvement in mood, anxiety, quality of life, and caregiver burden.
- **1C:** Establish recruitment and retention rates for this trial design.

##### Power and sample size

In our analyses of the imaging measures over the six-month intervention period, we expect that treatments will reduce age-dependent changes, measured as percent reduction in change from baseline, i.e., effect size (Cohen’s d). Using pilot data, we calculated means and standard deviations (SD) of changes in imaging variables as estimates for the non-treatment group. As in Aim 1, roughly 120 participants per group would be needed to detect an effect size of 0.325 with 80% power and α = 0.05. These effect sizes correspond to reductions in age-related imaging changes of roughly 30% or 50%. The present trial will also allow us to begin building towards those trial sizes.

#### Aim 2

To estimate effect sizes regarding the impact of computerized cognitive training, yoga, or an active control (wellness education) delivered in a multicomponent program that provides memory compensation and support groups to all subjects on cognitive function, daily function, mood, anxiety, and quality of life in persons with aMCI. We hypothesize that cognitive function, daily function, mood, anxiety, and quality of life will be higher in the computerized cognitive training and the yoga groups than in the active control group.

##### Power and sample size

Meta-analysis of studies of cognitive impact in healthy older adults suggest Cohen’s d within groups was 0.325 for aerobic exercise and 0.327 for cognitive training [18]. A power analysis in Gpower assuming two independent means indicated 118 people per arm are necessary to have 80% power to detect this magnitude of a difference between samples. Due to the group-randomized nature of the current trial, it is possible that a higher n is required. The scope of this pilot funding opportunity limits us to just 25 aMCI patients and their 25 partners per arm, leading to a total of 75 aMCI patients and their 75 partners. The results of the current trial may serve as pilot data for future studies.

## Methods

### Setting

Potential trial participants will be recruited from the Memory Disorder Clinics, behavioral neurology and neuropsychology practices at the University of Florida, Mayo Clinic Jacksonville, and Tallahassee Memorial Hospital. There will be three program sessions at each site. In order to reach target sample size, the trial will be advertised in healthcare and elderly living communities, at information sessions in the community, as well as by local healthcare professionals. We will also utilize advertising via brochures, flyers, newspaper articles, or other means suggested through the University of Florida, Mayo Clinic Jacksonville, and Tallahassee Memorial Hospital as appropriate. Medical providers may also refer patients with aMCI to the program.

### Participants

Our target is to have a total of 75 couples, recruited approximately evenly across the three sites. Enrollment will happen in dyads (i.e., a person with aMCI and a healthy study partner). Twenty-five dyads will be assigned to each treatment arm. Once written informed consent is obtained, participants will be screened for their eligibility over the phone. The Clinical Dementia Rating scale (CDR) [19] will be used to confirm the aMCI status of the patient and the Telephone Interview for Cognitive Status for Memory (TICS-M) will be used to confirm study partners are cognitively healthy. Specific inclusion and exclusion criteria are outlined in Table 1.

**Table 1:** Inclusion and exclusion criteria

Inclusion criteria	Exclusion criteria
For the person with aMCI: 1. Written informed consent for participation. 2. A formal diagnosis of amnestic mild cognitive impairment [1] (single domain or multidomain) in the last 6 months (by neuropsychological evaluation) OR A CDR score of 0 or 0.5 and a TICS-M score of at least 25 on the TICS-M. 3. Aged at least 50 years. 4. Either not taking or stable on nootropic(s) and/or pain medication on a dose and frequency that affects cognitive abilities for at least 3 months. 5. Fluent in English. For the study partner: 6. Written informed consent for participation. 7. Aged at least 21 years. 8. A score of at least 32 on the TICS-M 9. Study partner has at least twice-weekly contact with the participant.	For the person with aMCI: 1. MRI contraindications (e.g. ferrous metal in the body, claustrophobia, pregnancy) For both the person with aMCI patient and study partner: 2. Physical impairments, language comprehension deficits, or significant hearing disturbances that would limit ability to perform tasks or participate in the intervention

### Randomization and blinding

**Fig. 1:**
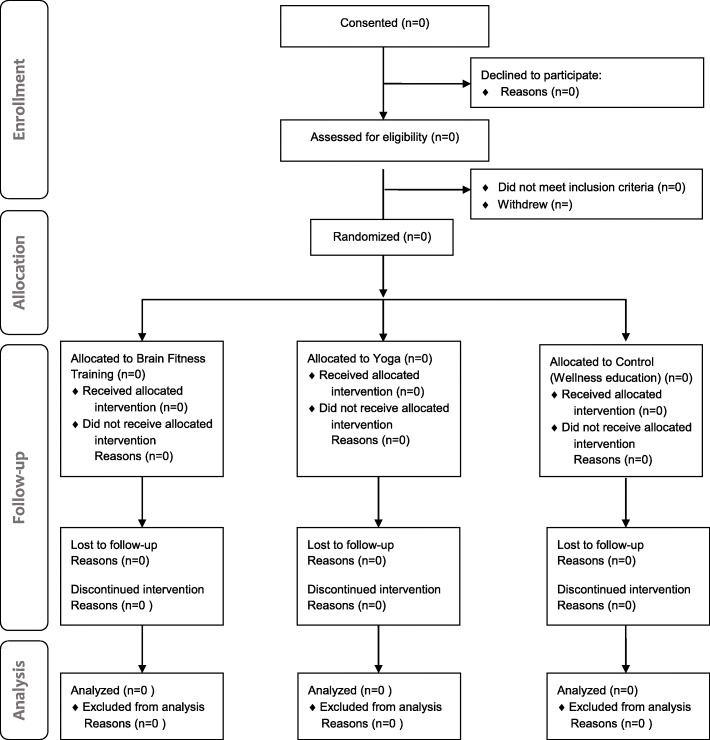
*Flow chart* showing summary of the pilot trial design for the Physical Exercise And Cognitive Engagement Outcomes For Mild Neurocognitive Disorder (PEACEOFMND) Trial. Adapted from CONSORT Flowchart

The group nature of intervention allows participants to compare their experiences, so individualized randomization poses a risk for diffusion of treatment effects. However, the group format limits in the ability to randomize per couple. Thus, we will block randomize by group instead of by participant assuring equal randomization of sessions to each of the three arms of the trial at each site. In other words, everyone in the first intervention period will receive the same treatment determined at random. The next intervention group will receive one of the two remaining arms also determined at random. The study biostatistician will randomize using a computerized random number generator to determine initial and second sessions at each site. The biostatistician is not involved in recruitment, assessment, or intervention delivery in any way. Due to the nature of the intervention, participants cannot be blinded. However, to mitigate selection bias, patients as well as study staff will be kept blind until recruitment for each session has been completed. Sessions will be offered sequentially three times in year 1 and include 6–12 individuals with aMCI and study partner couples per session. Hence, there will be the opportunity to block randomize nine (three sites × 3) sessions involving approximately 25 couples per arm for a total of 75 participants. A flow chart diagram showing summary of the pilot trial design can be found in Fig. 1.

### The intervention program

All participants in each arm of the intervention will receive memory compensation training and support groups in addition to the randomized intervention. During memory compensation training, the patient is trained to use a calendar and note-taking system in a consistent way in order to form calendar use habits [6, 7]. The use of the calendar is theorized to build primarily on procedural memory and priming, two types of implicit memory that remain relatively intact in aMCI. Separate support groups will be conducted with participants and study partners. A therapist will lead a discussion with regards to sharing disease-related experiences. There is no evidence to suggest and no theoretical reason to believe these compensatory/supportive interventions will impact the neuroimaging or cognitive measures in this trial. Even if they do, this effect should be balanced across the three arms of the interventions, as our active control group will also be receiving these treatments. In addition to these two “base” interventions, groups will be randomized to receive 60 min of daily yoga, computerized cognitive training, or wellness education. Each of these three interventions is described in more detail below:

- Yoga: Certified yoga instructors will use adapted hatha yoga where participants sit on armless chairs placed on sticky mats for some asana (poses) and use the chair for support throughout. This adapted hatha yoga style is appropriate for older adults including those who have limited mobility, use walkers, or are in wheelchairs. The appropriately sequenced yoga practice meets the American College of Sports Medicine recommendation for older adults for muscle strengthening and flexibility. Instructions are modeled for the participants.
- Computerized cognitive training: Use of a commercially available product, BrainHQ™, with scientific support for its efficacy in improving cognitive function. [20, 21]. We will use six validated modules from this program (Hawkeye, Divided Attention, Double Decision, Sound Sweeps, Syllable Stacks, and Memory Grid). Study staff will help participants with navigating the program. After the initial two-week intervention period, participants’ adherence and progress will be tracked through the computerized cognitive training portal.
- Active control (wellness education): In order to control for contact times and the calendar and support group interventions, controls will receive wellness education by licensed psychologists focused on the importance of diet, sleep hygiene, social engagement, and physical exercise delivered in a similar fashion to the other interventions.

### Intervention timeline

**Fig. 2:**
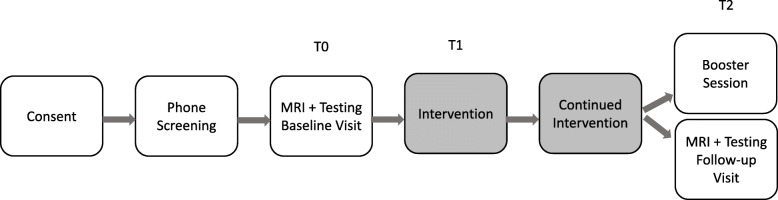
Participant timeline

There will be nine different intervention groups, each starting at a different point in time. The initial intervention period will consist of 1 h daily of the assigned intervention (yoga, computerized cognitive training, or wellness education) in combination with 1 h of memory compensation training and 1 h of support groups. This will last for a period of ten days, delivered weekdays over two weeks. After the initial two-week intervention, participants will continue the intervention they were assigned to (yoga or computerized brain fitness) for another 24 weeks. Participants need to engage in the assigned intervention for at least 2 h for at least 20 out of 24 weeks to be considered a program completer. They are encouraged to do more. Attendance of the weekly yoga session as well as the number of minutes spent on yoga at home will be recorded each week. Individuals who miss a yoga session will be called by the study staff with the goals to record the number of minutes spent on yoga at home and will be encouraged to attend the next yoga session. For the computerized cognitive training arm, staff will track progress solely through the BrainHQ™ portal on a weekly basis. Individuals who spend < 120 min on computerized cognitive training will be contacted by the study staff and will be encouraged to spend more time on computerized cognitive training next week. The wellness education group will be asked to fill out and submit wellness logs for sleep, nutrition, and mood each week for 24 weeks. Individuals who do not send in their weekly log will be contacted via phone by the study staff and will be encouraged to send in their log next week. The amount of phone calls of the study coordinators to the participants will be recorded. Participants will not be restricted in concomitant care and interventions. A timeline chart from the perspective of participants is shown in Fig. 2.

### Discontinuation

Discontinuation of participants will occur upon participants’ request. If a participant discontinues, no new data on this participant will be collected.

### Outcome measures

**Fig. 3:**
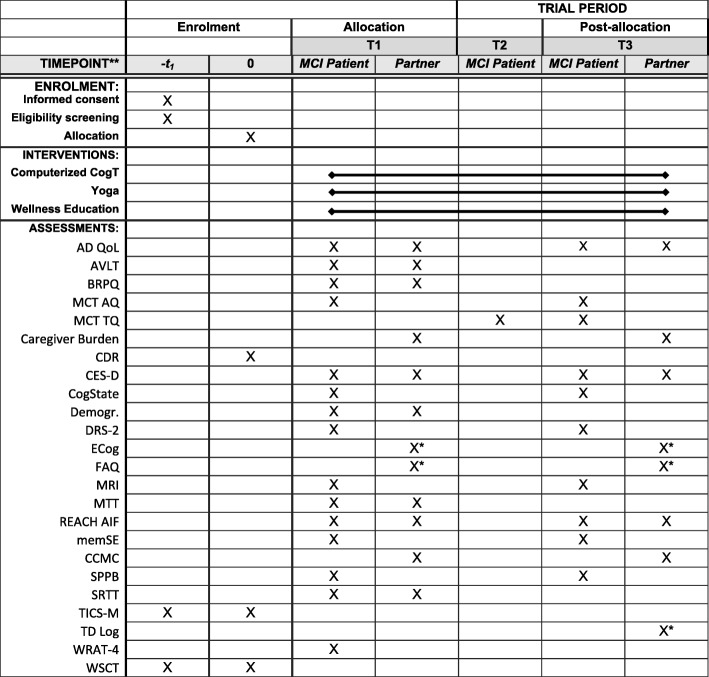
Schedule of enrolment, interventions, and assessment. Adapted from SPIRIT figure 2013

An overview of all measures in given in Fig. 3.

#### Cognitive functioning

- The Dementia Ratings Scale-2 (DRS-2) [22] will be used at baseline to asses overall level of cognitive functioning and after the six-month intervention period to monitor cognition during the six-month intervention period in the persons with aMCI. The DRS-2 assesses multiple cognitive domains and helps to distinguish individuals with aMCI from patients with Alzheimer’s dementia and healthy controls [23].
- The CogState, a computerized cognitive assessment, will be used to monitor more specific cognitive domains at baseline and after the six-month intervention period in the person with aMCI. CogState is sensitive to cognitive aging effects in MCI and dementia and therefore to interventions that remediate these effects [24]. The following subtasks of the CogState will be used: Detection task – a simple reaction time paradigm that measures psychomotor function; Identification task – a choice reaction time paradigm that measures visual attention; One Card Learning task – a continuous visual recognition learning task that assesses visual recognition memory and attention; One Back task – a task that assesses working memory and attention.
- The Rey Auditory Verbal Learning Test (RAVLT) [25, 26] will be administered to assess declarative memory. A 15-word list will be read to the participants, after which they will be asked to repeat all words they remember. This will be done five times. After 30 min, a delayed recall and recognition trial will be conducted.
- The Word Stem Completion Test (WSCT) [27] will be administered to assess priming memory. A word completion method similar to the method used in previous studies [28, 29] will be used.
- The Mirror-Tracing Task (MTT) [30] will be used to asses procedural memory. Participants are asked to trace the cutout outline of a five-point star without touching the edges of the star while looking in a mirror. Their direct view of their own hand and the figure to be traced will be blocked. Testing consists of two blocks of five trials, separated by an interval of 15 min.
- The Serial Reaction Time Task (SRTT) will be administered as a procedural memory task that involves only a limited motor component. The SRTT was developed largely based on the SRTT used in previous research [31] and will be administered on an Android-run tablet (Samsung SM-T580NZWAXAR 10.1 Galaxy Tab A T580) using OpenSesame Software [32].
- At baseline, the Wide Range Achievement Test 4 (WRAT4) [33] reading subtest will be administered to assess the patients’ reading achievement and cognitive reserve. Participants read aloud a list of 55 words that increase in complexity throughout the task and 1 point is awarded for each correctly pronounced word. Previous studies have successfully utilized reading ability as a measure of cognitive reserve [34].

#### Functional status

All measures of functional status will be conducted at baseline and after the six-month intervention period.

- The study partners will be asked to fill out the Everyday Cognition (E-Cog) [35] about the individuals with aMCI’s functioning in instrumental activities of daily living (IADLs). The E-Cog is an informant-based measure that assesses the participant’s ability to perform everyday tasks in the following areas: memory; language; visuospatial abilities; planning; organization; and divided attention.
- The study partners will be asked to fill out the Functional Assessment Questionnaire (FAQ) [36] about the person with aMCI’s functional status. The FAQ is the standard functional measure required for use throughout the Alzheimer’s Disease Research Center network.
- The study partners will be asked to fill out the Treatment Diffusion Log about the person with aMCI’s and their own time spent on any type of physical exercise, computerized cognitive training, wellness behaviors, use of a calendar, and supportive therapy. This will be done to assess specific skills targeted by the interventions.
- The Memory Compensation Training Therapists will fill out the MCT Adherence Questionnaire about the person with MCI’s use of the calendar. This questionnaire assesses how well an individual utilizes the sections of the MCT calendar system. The evaluator will examine MCT compliance for two randomly selected days from the prior week. This will be done on the first day of treatment, as well as during the booster session.
- The Memory Compensation Training Therapists will also be asked to determine the MCT Training Score on the final day of the program This 0–6 score reflects the individual with MCI’s progress in the three phases of calendar training. A score of 0 reflects no progress while a score of 6 reflects mastery of all three training phases. Intermediate scores (1–5) reflect intermediate levels of training progress.
- The Short Physical Performance Battery (SPPB) [37] will be administered to the individuals with aMCI to monitor their physical performance. This includes a timed 8-ft walk, standing side by side, semi-tandem and full-tandem stance, and a timed arms-folded rise from seated to standing.

#### Quality of life, mood, and anxiety

The quality of life, mood, and anxiety measures will be conducted at baseline and after the six-month intervention period.

- Both the persons with aMCI and the study partners will be asked to complete the Quality of Life-AD (AD-QOL) [38] at baseline and after six months. The AD-QOL is a 13-item measure developed for individuals with dementia that has been utilized in aMCI and with study partners. Individuals with aMCI and study partners rate their relationships, concerns about finances, physical condition, mood, energy level, memory, aspects of daily functioning, and overall life quality on a four-point scale.
- Both individuals with aMCI and study partners will be asked to complete the Center for Epidemiologic Studies Depression Scale (CES-D), a well validated 20-item self-report measure of depressive symptoms with a four-factor structure consisting of negative affect, lack of positive affect, somatic symptoms, and interpersonal difficulties subscales [39].
- Both the persons with aMCI and the study partners will complete the REACH Anxiety Inventory Form (REACH-AIF) [40], a 10-item rating scale modified from the State-Trait Anxiety Inventory, by the Resources for Enhancing Alzheimer’s Caregiver Health (REACH) project [41].

#### Self-efficacy

- The persons with aMCI will be asked to complete selected (based on their relevance to memory-based daily activities) items from the Chronic Disease Self-Efficacy Scales [42]. The language from the original scales was modified to be specific to memory (i.e. “your memory/cognition” rather than more general references to “your health condition”). The result is the nine-item Self-Efficacy in Memory in MCI scale (memSE).
- Study partners will be asked to complete the Caregiving Competence and Mastery Components (CCMC) of the Pearlin [43]. These measures reflect their titles and are in the range of 4–6 items.

#### Attitudes towards research

- Barriers to Research Participation Questionnaire (BRPQ) [44]: Both the persons with aMCI and the study partners will be asked to complete the BRPQ at baseline. BRPQ is a 17-item screener that assesses attitudes towards research participation. This scale assesses five predictors of study participation (i.e. mistrust, religious beliefs, health beliefs/fears, role overload/time demands, and perceived personal and community benefits).

#### MRI acquisitions

All images will be acquired using a 3-T MRI scanner (Siemens Prisma) that is controlled for quality and are monitored weekly. We will use imaging to assess the efficacy and to elucidate the mechanisms of intervention impact by measuring brain changes over the intervention period. The primary imaging outcomes will be structural MRI (sMRI), arterial spin labeling (ASL), and resting state fMRI. Diffusion tensor imaging (DTI) will also be acquired. In addition, we will also ascertain the degree of vascular pathology using fluid-attenuated inversion recovery (FLAIR) and susceptibility-weighted MRI. The detailed imaging protocol and imaging measures are described in the supplementary neuroimaging protocol, which can be found in Additional file 1.

### Data management

An online REDCap database will be used, supported by Mayo Clinic Jacksonville. To ensure high-quality data, we will use double-data entry for 20% of the participant couples, randomly selected at each site. If data entry consistency is < 95%, all data will be entered using double-data entry. There will also be a range check in place for each measure.

### Statistical methods

The primary analyses will use linear mixed effects models to examine change from baseline to post intervention (~ 6 months) in each of the outcomes. These models control for within-subject effects through the use of per-subject random effects for slopes and intercepts over the six-month follow-up period. We will estimate the degree of change over time in each of the specific measurement intervals within randomized groups and test for differences in these changes between groups while conforming to linear model assumptions. We will evaluate the assumptions required for the appropriate use of linear models and will apply any needed transformations in the data to conform to the model assumptions. Analyses will be conducted to assess the aims listed above. In addition, to make it possible to identify which individuals are most likely to benefit from the interventions, we will incorporate variables to evaluate whether variables either attenuate or modify cognitive or imaging changes. Additional analyses are currently unknown. Missing data will be handled based on what appears to be most appropriate for the analysis being conducted. No interim analyses will be conducted.

### Data monitoring

The principle investigator (PI) at each site will be responsible for monitoring the safety and efficacy of this pilot trial and executing the Data and Safety Monitoring Plan (DSMP). The DSMP will be reviewed on a monthly basis by the PI and other co-investigators as necessary.

### Harms

In this trial, we will use the Food and Drug Administration's definition of serious adverse events (SAEs) and adverse events (AEs). SAEs are unlikely in these behavioral interventions. Any SAE, whether or not it is related to the intervention, will be reported to the site Institution Review Board (IRB). The PI at each site will monitor for the presence of both SAEs and AEs at each scheduled visit. These individuals will report any SAE or AE to the overall PI twice a month. However, if the SAE involves death or a life-threatening event, the site PI will be notified within 24 h and the PI will notify the IRB within two working days from the time the SAE was first reported. Reports of SAEs received by the IRB will be reviewed by an institutional SAE board to determine the seriousness of the event and what actions, if any, will be required. In the event that a participant withdraws from the trial or the PI decides to discontinue a participant due to an SAE, the participant will be monitored by the site PI via ongoing status assessment until: (1) resolution of the problem is reached; or (2) the SAE is determined to be clearly unrelated to the trial intervention. Summary and outcome of all SAEs will be reported to the IRB annually.

### Auditing

There are no planned auditing procedures for trial conduct in place for the current trial.

### Protocol amendments

Important protocol modifications will be communicated to each of the authors and other relevant study-investigators and will be updated on ClinicalTrials.gov. IRB approval will be obtained before any important protocol modifications will be implemented. Participants will be notified should there be any important protocol modifications that would concern them.

### Confidentiality

Only the PI and Sub-Investigators that are approved by any of the three IRBs (of the University of Florida, Mayo Jacksonville, and Tallahassee Memorial Healthcare), other professionals at the study site that provide trial-related treatment or procedures, and the three IRBs are allowed to collect, use, and share protected health information. Once collected, protected health information may be shared with the study sponsor, United States governmental agencies who are responsible for overseeing research such as the Food and Drug Administration, the Department of Health and Human Services and the Office of Human Research Protections, government agencies who are responsible for overseeing public health concerns such as the Centers for Disease Control and federal, state, and local health departments, participants’ insurance companies for purposes of obtaining payment for the Support Groups and Memory Compensation components of the trial, and between the IRB-approved investigators of the other study sites (between the University of Florida, Mayo Jacksonville and Tallahassee Memorial Healthcare). Otherwise, no protected health information will be released without permission of the participant unless required by law or a court order. After the trial has been completed, protected health information will no longer be shared and used; it will be coded and will become part of a research database.

### Ancillary and post-trial care

In the event of injury as a result of study participation, the Mayo Clinic, Tallahassee Memorial Hospital, or the University of Florida will provide medical services for treatment. Such services will be provided for free if not covered by a health plan or insurance. No additional compensation will be given.

### Dissemination policy

The results will be presented at both national and international conferences and published in topline journals. Results will also be shared with participants upon request. If any questions arise regarding the data, the protocol, and the statistical codes, any of the PIs may be contacted.

## Discussion

Computerized cognitive training and increased physical activity have shown to be amongst the most promising interventions in individuals with aMCI. However, the neural mechanisms underlying these interventions are currently unclear. The aim of this pilot trial is to study the feasibility of an investigation of the neuroimaging impact of computerized cognitive training versus yoga versus an active control intervention, as part of a multi-component treatment program, in persons with aMCI. Strengths of the current trial include the multicomponent design, which allows us to compare treatments while all participants received an intervention. The limitations of the current trial include our inability to blind participants to their experimental condition, possibly biasing results. Our Treatment Diffusion log was developed to control for this bias. The current trial will lay the groundwork for a larger study examining the impact of computerized cognitive training, yoga, and an active control intervention (wellness education) on changes in cerebral perfusion and functional connectivity as well as cognition, daily functioning, mood, anxiety, and quality of life in individuals with aMCI. The pilot trial will be conducted and reported in accordance with the Consolidated Standards of Reporting Trials (CONSORT) and the Standard Protocol Items: Recommendations for Interventional Trials (SPIRIT) guidelines. In Additional file 2, the SPIRIT 2013 Checklist is provided. In Additional file 3 and 4, the CONSORT 2010 guidelines checklists for reporting a pilot or feasibility trial and for journal or conference abstracts are provided.

## Trial status

At the time of submission of this paper, patient recruitment is still ongoing.

## Additional files

Additional file 1: Supplementary neuroimaging protocol. (PDF 66 kb)

Additional file 2: SPIRIT 2013 Checklist: Recommended items to address in a clinical trial protocol and related documents. (PDF 113 kb)

Additional file 3: CONSORT 2010 checklist of information to include when reporting a pilot or feasibility trial. (PDF 216 kb)

Additional file 4: CONSORT 2010 checklist of information to include when reporting a pilot or feasibility randomized trial in a journal or conference abstract. (PDF 189 kb)

## Abbreviations

AD QoL: Quality of Life-AD
ADLs: Activities of daily living
AE: Adverse event
aMCI: Amnestic mild cognitive impairment
BRPQ: Barriers to Research Participation Questionnaire
CCMC: Caregiving Competence and Mastery Components of the Pearlin
CDR: Clinical Dementia Rating scale
CES-D: Center for Epidemiologic Studies Depression Scale
DRS-2: Dementia Rating Scale-2
DSMP: Data and Safety Monitoring Plan
DTI: Diffusion tensor imaging
E-Cog: Everyday Cognition
FAQ: Functional Assessment Questionnaire
FLAIR: Fluid-attenuated inversion recovery
HABIT: Healthy Action to Benefit Independence and Thinking
IRB: Institution Review Board
LAR: Legally authorized representative
memSE: Self-Efficacy in Memory scale
MRI: Magnetic resonance imaging
MTT: Mirror-tracing task
PEACEOFMND: Physical Exercise And Cognitive Engagement Outcomes for Mild Neurocognitive Disorder
PI: Principle investigator
RAVLT: Rey Auditory Verbal Learning Test
REACH AIF: Anxiety Inventory Form by the Resources for Enhancing Alzheimer’s Caregiver Health project
SAE: Serious adverse event
sMRI: Structural magnetic resonance imaging
SPPB: Short physical performance battery
SRTT: Serial reaction time task
TD Log: Treatment Diffusion Log
TICS-M: Telephone Interview for Cognitive Status for Memory
WRAT4: Wide Range Achievement Test 4
WSCT: Word stem completion test
