# Supplementary material for: Physical exercise and cognitive engagement outcomes for mild neurocognitive disorder: a group-randomized pilot trial
Source: Trials. 2018 Oct 19;19:573. doi: 10.1186/s13063-018-2865-3 (PMC6194559; doi:10.1186/s13063-018-2865-3)
Supplement: Supplementary file 1 — Supplementary neuroimaging protocol. (PDF 66 kb) [file 13063_2018_2865_MOESM1_ESM.pdf]

Supplement 1. Supplementary Neuroimaging Protocol

MRI acquisitions will be conducted using a 3T Siemens Prisma. The 3T Siemens Prisma whole body scanner includes simultaneous multi-slice (SMS) techniques for functional MRI (fMRI) and diffusion imaging (dMRI) as outlined in the Human Connectome Project. The Siemens Prisma includes an 80 mT/m @ 200 T/m/s gradient system, parallel transmit capabilities, and a series of MR coils including a 64-channel head/neck coil used for the investigation. Information that is more specific can be found in Table S1.

Table S1. Neuroimaging specifics.

**Sequence Name**	**FOV and resolution in mm**	**Parameters (ms)**	**Run time in minutes**	**Purpose**	**Notes**
**MP-RAGE**	Sagittal: 256x256x208 (1x1x1 mm)	TR=2000, TE=2.91, TI=1010, Flip angle=8°	3:33	T1-weighted structural analyses, lacunar infarction quantification	
**T2**	Sagittal: 256x256x208 (1x1x1 mm)	TR=2500, TE=370, Flip angle=120°	4:15	T2-weighted structural analyses (combined with and independent from T1)	
**3D T2 FLAIR**	Sagittal: 256x256x176 (1x1x1 mm)	TR=4800, TE=441, TI=1650, Flip angle=120°	6:58	Quantification of white matter disease	
**SWI**	Axial: 256x256x128 (1x1x1 mm)	TR=35, TE=15, Flip angle=12°	5:35	Analysis of hemorrhage/blood products, calcium, and iron	
**Resting state fMRI**	Axial: 80x80x43 (3x3x3 mm)	TR=3500, TE=30, Flip angle=80°	10:00	Functional connectivity analyses	
**pASL**	Axial: 64x64x9 (4x4x8)	TR=2500, TE=12, Flip angle=90°	3:52	Metabolism and perfusion analyses	
**DWI**	Axial: 128x128x197 (2x2x2 mm)	TR=3200, TE=70, Flip angle=90°	10:52 (plus 0:33 for each additional AP and PA b0 images)	Structural connectivity analyses	AP and PA phase reversed b0 images also acquired to assist in distortion correction. SMS = 3, Multi-shell acquisition: b = 0, 1000, 2000, 3000

Abbreviations: DWI: Diffusion Weighted Imaging; fMRI: functional Magnetic Resonance Imaging; FLAIR: Fluid Attenuated Inversion Recovery; FOV: field-of-view; mm: millimeters; ms: milliseconds; MP-RAGE: magnetization prepared rapid gradient-echo; pASL: pulsed Arterial Spin Labeling; TE: Echo Time; TI: Inversion Time; TR: Repetition Time; SMS: simultaneous multi-slice; SWI: susceptibility weighted imaging.
