# Supplementary material for: Physical exercise and cognitive engagement outcomes for mild neurocognitive disorder: a group-randomized pilot trial
Source: Trials. 2018 Oct 19;19:573. doi: 10.1186/s13063-018-2865-3 (PMC6194559; doi:10.1186/s13063-018-2865-3)
Supplement: Supplementary file 4 — CONSORT 2010 checklist of information to include when reporting a pilot or feasibility randomized trial in a journal or conference abstract. (PDF 189 kb) [file 13063_2018_2865_MOESM4_ESM.pdf]

CONSORT 2010 checklist of information to include when reporting a pilot or feasibility randomized trial in a journal or conference abstract

**Item**	**Description**	**Reported on line number**
Title	Identification of study as randomised pilot or feasibility trial	1
Authors *	Contact details for the corresponding author	38
Trial design	Description of pilot trial design (eg, parallel, cluster)	8
Methods		
Participants	Eligibility criteria for participants and the settings where the pilot trial was conducted	11
Interventions	Interventions intended for each group	11-12
Objective	Specific objectives of the pilot trial	6-10
Outcome	Prespecified assessment or measurement to address the pilot trial objectives**	18
Randomization	How participants were allocated to interventions	14
Blinding (masking)	Whether or not participants, care givers, and those assessing the outcomes were blinded to group assignment	14-15
Results		
Numbers randomized	Number of participants screened and randomised to each group for the pilot trial objectives**	10-11
Recruitment	Trial status†	
Numbers analysed	Number of participants analysed in each group for the pilot objectives**	NA
Outcome	Results for the pilot objectives, including any expressions of uncertainty**	NA
Harms	Important adverse events or side effects	NA
Conclusions	General interpretation of the results of pilot trial and their implications for the future definitive trial	NA
Trial registration	Registration number for pilot trial and name of trial register	22
Funding	Source of funding for pilot trial	

By Eldridge SM, Chan CL, Campbell MJ, Bond CM, Hopewell S, Thabane L, et al. CONSORT 2010 statement: extension to randomised pilot and feasibility trials. BMJ. 2016;355.
